# Knee temperature remains abnormal in patients successfully treated with anterior cruciate ligament reconstruction: An infrared thermography analysis

**DOI:** 10.1002/jeo2.70012

**Published:** 2024-09-09

**Authors:** Luca De Marziani, Simone Orazi, Angelo Boffa, Luca Andriolo, Alessandro Di Martino, Stefano Zaffagnini, Giuseppe Filardo

**Affiliations:** ^1^ Clinica Ortopedica e Traumatologica 2 IRCCS Istituto Ortopedico Rizzoli Bologna Italy; ^2^ Applied and Translational Research (ATR) Center IRCCS Istituto Ortopedico Rizzoli Bologna Italy; ^3^ Faculty of Biomedical Sciences Università della Svizzera Italiana Lugano Switzerland

**Keywords:** ACL, anterior cruciate ligament reconstruction, inflammation, infrared thermography, knee

## Abstract

**Purpose:**

The aim of this study was to evaluate if the operated knee environment remains abnormal in patients successfully treated with anterior cruciate ligament reconstruction (ACL‐R).

**Methods:**

Thirty asymptomatic patients were enrolled (28 men, 2 women, age 28.6 ± 6.54 years, body mass index: 24.9 ± 3.0 kg/m^2^) and evaluated at 42.2 ± 12.5 months after surgery. Patients were assessed with patient‐reported outcome measurements and with a triaxial accelerometer. The temperature of the knees as well as four regions of interest were evaluated with an infrared thermographic camera FLIR T1020 (FLIR® Systems) according to a standardised protocol including a baseline evaluation and further evaluations immediately after exercise and after 5, 10 and 20 min. The temperature of the ACL‐R knee was compared to that of the contralateral healthy knee for the purpose of the study.

**Results:**

The mean temperature of the knee was higher (*p* = 0.010) for the ACL‐R knees (31.4 ± 1.4°C) compared to the healthy knees (31.1 ± 1.6°C), as well as for the patellar area (*p* = 0.005), the lateral area (*p* = 0.016) and the medial area (*p* = 0.014). The analysis of the response to the exercises of the ACL‐R knees showed similar trends to the healthy knees but higher temperature values at all time points (*p* < 0.05). Patients who underwent ACL‐R with concomitant meniscal treatment showed higher knee temperatures compared to ACL‐R knees without concomitant meniscal treatment after 5 (*p* = 0.047), 10 (*p* = 0.027) and 20 min (*p* = 0.048).

**Conclusions:**

The temperature of asymptomatic knees previously treated with ACL‐R is higher than the contralateral healthy knee, both at rest and after exercise, with a further increase in knees that underwent both ACL‐R and meniscal treatment. These results suggest an inflammatory state persisting years after the surgery, which could predispose to the early onset of knee degeneration.

**Level of Evidence:**

III, Case–control study.

**Abbreviations**
ACL: anterior cruciate ligament
ACL‐R: anterior cruciate ligament reconstruction;
BMI: body mass index
EQ‐VAS: EuroQol Visual Analogue Scale
IKDC: International Knee Documentation Committee
KOOS: Knee injury and Osteoarthritis Outcome Score
OA: osteoarthritis
PROM: patient‐reported outcome measurement
ROI: region of interest
VAS: visual analogue scale

## INTRODUCTION

Anterior cruciate ligament (ACL) treatment results improved over the years, leading to overall satisfying results both in terms of symptom resolution and functional activity recovery [9, 25, 63]. Nonetheless, an increased risk of early knee osteoarthritis (OA) can remain [1, 11]. The presence of cartilage lesions as well as concurrent or subsequent meniscal lesions are considered possible aspects leading to a worse outcome and joint degeneration [21]. Moreover, the ACL injury triggers an increase of inflammatory cytokines in the joint [38] and ACL reconstruction (ACL‐R) constitutes a further insult to the already compromised joint [38]. While the specific reasons leading to the high prevalence of joint degeneration remain debated and are likely multifactorial, detecting early stages of joint environment abnormalities could be useful to understand the evolution of ACL reconstructed knees, and possibly to better manage these patients based on their joint status.

Infrared thermography is a valuable method for non‐invasively assessing body temperature, enabling the evaluation of specific thermal patterns in body parts that could be affected by conditions activating the inflammatory cascade [47]. Recent technical advancements led to extended thermography use across several medical fields, ranging from wound and ulcer monitoring to breast cancer screening [45]. In orthopaedics, thermography has proven to be a useful tool for studying musculoskeletal pathologies, such as tendon disorders, inflammatory joint conditions, and even infection in total knee arthroplasty [16, 42, 46, 50, 57, 59]. Previous studies demonstrated thermography's ability to highlight joint inflammation and early knee OA stages [13, 15]. However, there is currently no evidence on thermographic patterns of knees successfully treated with ACL‐R. The assessment of knees treated with ACL‐R using infrared thermography could be of clinical relevance by detecting an increase in temperature due to underlying inflammation, bringing to light a possibly neglected inflammatory process that could potentially contribute to early onset knee OA.

The aim of this study was to evaluate patients successfully treated with ACL‐R to investigate if the operated knee environment remains abnormal as detected by a temperature increase using infrared thermography. The hypothesis of this study was that the joint environment remains altered even in young asymptomatic patients successfully treated with ACL‐R.

## METHODS

### Study design and selection criteria

Male or female patients aged between 18 and 40, with a previous unilateral ACL‐R between 2 and 5 years before the evaluation, without a history of subjective instability or pain in the treated knee after surgery, with a subjective International Knee Documentation Committee (IKDC) value ≥90, and with a contralateral healthy knee (no history of trauma or previous surgery and a visual analogue scale (VAS) for pain = 0) were included in the study. The following exclusion criteria were used: body mass index (BMI) <18.5 or >30 kg/m^2^, presence of neoplasms, dermatological and vascular conditions, metabolic disorders of the thyroid, cardiovascular diseases, rheumatoid arthritis, inflammatory arthropathy, haematological diseases, infections, immunodepression, antidepressant, anticoagulants or antiaggregant therapy, and use of nonsteroidal anti‐inflammatory drugs in the 5 days before the investigation. Patients were recruited through an institutional announcement. A total of 102 patients received the information about the study. Among these patients, 72 were excluded because they did not match the inclusion/exclusion criteria.

### Ethical aspects

This study was approved by the local Ethics Committee of the IRCCS Istituto Ortopedico Rizzoli, Italy (no. 359/2023/Sper/IOR). Patients previously treated with ACL‐R were enroled and evaluated by orthopaedic physicians between July 2023 and February 2024 in a research outpatient clinic focused on sports medicine and knee osteoarthritis. Informed consent was obtained from each patient for study participation.

### Patients' characteristics and evaluation

Thirty consecutive patients previously treated with ACL‐R were enroled according to the inclusion/exclusion criteria and evaluated at a mean time after surgery of 42.2 ± 12.5 months. Among them, 2 were women and 28 were men, with a mean age of 28.6 ± 6.48 years and a mean BMI of 24.9 ± 3.0 Kg/m^2^. After enrolment in the study and before the infrared thermography acquisition of the ACL‐R and contralateral healthy knee, patients were clinically evaluated through knee‐specific patient‐reported outcome measurements (PROMs) including the IKDC subjective and objective scores [29], the Marx Activity Rating Scale [40], the Knee injury and Osteoarthritis Outcome Score (KOOS) sub‐scales [48], the VAS for pain, the EuroQol Visual Analogue Scale (EQ‐VAS) [20] and the Tegner score [58]. Subjective clinical questionnaires were compiled by patients with the support of the clinician, while the IKDC objective score was evaluated by the clinician. In addition, knee laxity differences between the ACL‐R knee and contralateral healthy knee were assessed in all patients through the Lachman and pivot shift tests using a triaxial accelerometer, the Kinematic Rapid Assessment (KiRA– OrthoKey). Finally, the temperature of the knees was evaluated with thermography imaging as reported below. All demographic and clinical patients’ characteristics are reported in Table 1.

**Table 1:** Included patients' characteristics.

Demographic characteristics	
Sex (W/M)	2/28
Age (years)	28.6 ± 6.4
BMI (kg/m^2^)	24.9 ± 3.0
Smoke (yes/no)	14/16
Time injury to surgery (months)	13.4 ± 11.1
Time after surgery (months)	42.2 ± 12.5
Concomitant meniscal tear (yes/no)	20/10
Meniscal treatment	Meniscal repair: 18 Partial meniscectomy: 2

Clinical characteristics	
IKDC Subjective score	95.1 ± 3.4
IKDC Objective score	A: 30
VAS pain	0.1 ± 0.3
KOOS Pain	94.2 ± 6.5
KOOS Symptoms	84.5 ± 10.7
KOOS ADL	98.4 ± 2.8
KOOS QoL	89.9 ± 7.4
KOOS Sport/Rec	87.3 ± 12.3
Marx Activity Rating Scale	9.8 ± 4.9
Tegner score	5.6 ± 1.7

Laxity evaluation	
Lachman (mm) difference (ACL‐R vs. healthy knee)	0.5 ± 2.1
Pivot shift (m/s^2^) difference (ACL‐R vs. healthy knee)	0.1 ± 1.3

*Note*: Values are expressed as mean ± standard deviation.

Abbreviations: ACL‐R, anterior cruciate ligament reconstruction; ADL, activities of daily living; BMI, body mass index; IKDC, International Knee Documentation Committee; KOOS, Knee injury and Osteoarthritis Outcome Score; M, male; QoL, quality of life; Sport/Rec, function in sport and recreation; VAS, visual analogue scale; W, women.

### Infrared thermography procedure and analysis

Infrared imaging evaluation was acquired in a dedicated outpatient clinic shielded from direct sunlight and with a temperature controlled and set at 23.0°C and a mean humidity of 45 ± 3% [13]. In order to minimise the circadian variation of the temperature, image acquisition was always performed in the same time slot between 15:00 and 18:00. According to the American Academy of Thermology guidelines [52], patients were asked to sit for 15 min with light clothing on the top to speed up the thermalisation and without touching their knee before the thermal image acquisition. The participants were asked to stand on a designated floor map. The thermograms of knees were conducted using a thermographic camera FLIR T1020 (FLIR® Systems), which has 1024 × 768 pixels of resolution and a thermal sensitivity of 0.02°C. The camera was positioned at a 1‐m distance from the subject, positioned perpendicular to the knee and adjusted to their patellar height. An anterior view image was obtained for each patient using the autofocus modality (T0).

To explore the behaviour of the skin temperature of both ACL‐R and contralateral healthy knees in response to exercise, an alternating single‐leg squat exercise with a distance of 70 cm was performed for 2 min at the rate of one eccentric and one concentric phase of the single leg every 2 s (1 s eccentric phase and 1 s concentric phase). A metronome was used to standardise pacing. Immediately after performing this exercise, the patient was positioned on the floor map and a second anterior view image was acquired (T1). Afterwards, the patient waited in the room for 5 min in a sitting position without touching or moving the lower limbs. At the end of this resting period, the patient was positioned on the floor map and a third anterior view image was acquired (T2). The same protocol was executed 10 min (T3) and 20 min (T4) after the exercise. Finally, maintaining the same position of the knee, an anatomical marker (circular adhesive of 2 cm in diameter) was placed at the centre of the patella to obtain a further image in the anterior view to facilitate the precise subsequent location of the patella in the analysis of the previous infrared images.

For the image analysis process, the five anterior images acquired at T0, T1, T2, T3 and T4 were aligned side by side with the image with the patellar marker on the computer screen, and, using the marked image as a guide, a template indicating the regions of interest (ROIs) was centred over the patella of each unmarked image. The ROIs were defined as follows: the patellar area was a square of 6 cm in width centred on the patella, the ‘suprapatellar’ area was the area 3 cm over the patella; and the ‘medial’ and ‘lateral’ areas were the regions 3 cm under the patella and on its medial and lateral sides, respectively [13]. The sum of the four ROIs has been defined as the ‘total knee temperature’. The mean temperatures were extracted using the software ResearchIR (FLIR® Systems) for the overall knee area and for the four ROIs: patella, suprapatellar, medial and lateral.

The thermographic images obtained for both the ACL‐R and contralateral healthy knees were compared to analyse differences between the operated and non‐operated joints. Moreover, correlations between the thermographic images and patient and joint characteristics were investigated.

### Statistical analysis

All quantitative data were expressed in terms of the mean and the standard deviation of the mean; the categorical data were expressed as frequency and percentages. The Shapiro–Wilk test was performed to test the normality of continuous variables. The Levene test was used to assess the homogeneity of the variances. The Paired *T* test was performed to assess the temperature differences between the operated and non‐operated joints. The ANOVA repeated measures followed by the Sidak post hoc pairwise comparisons were performed to assess the temperature differences along the follow‐up after the exercises. One‐way ANOVA with the Scheffé post hoc pairwise analysis was performed to assess the temperature differences among the areas when the Levene test for homogeneity of variances was not significant (*p* < 0.05); otherwise, the Kruskal−Wallis test with the non‐parametric post hoc pairwise Dunnet test was used. For all tests, *p* < 0.05 was considered significant.

## RESULTS

**Figure 1:**
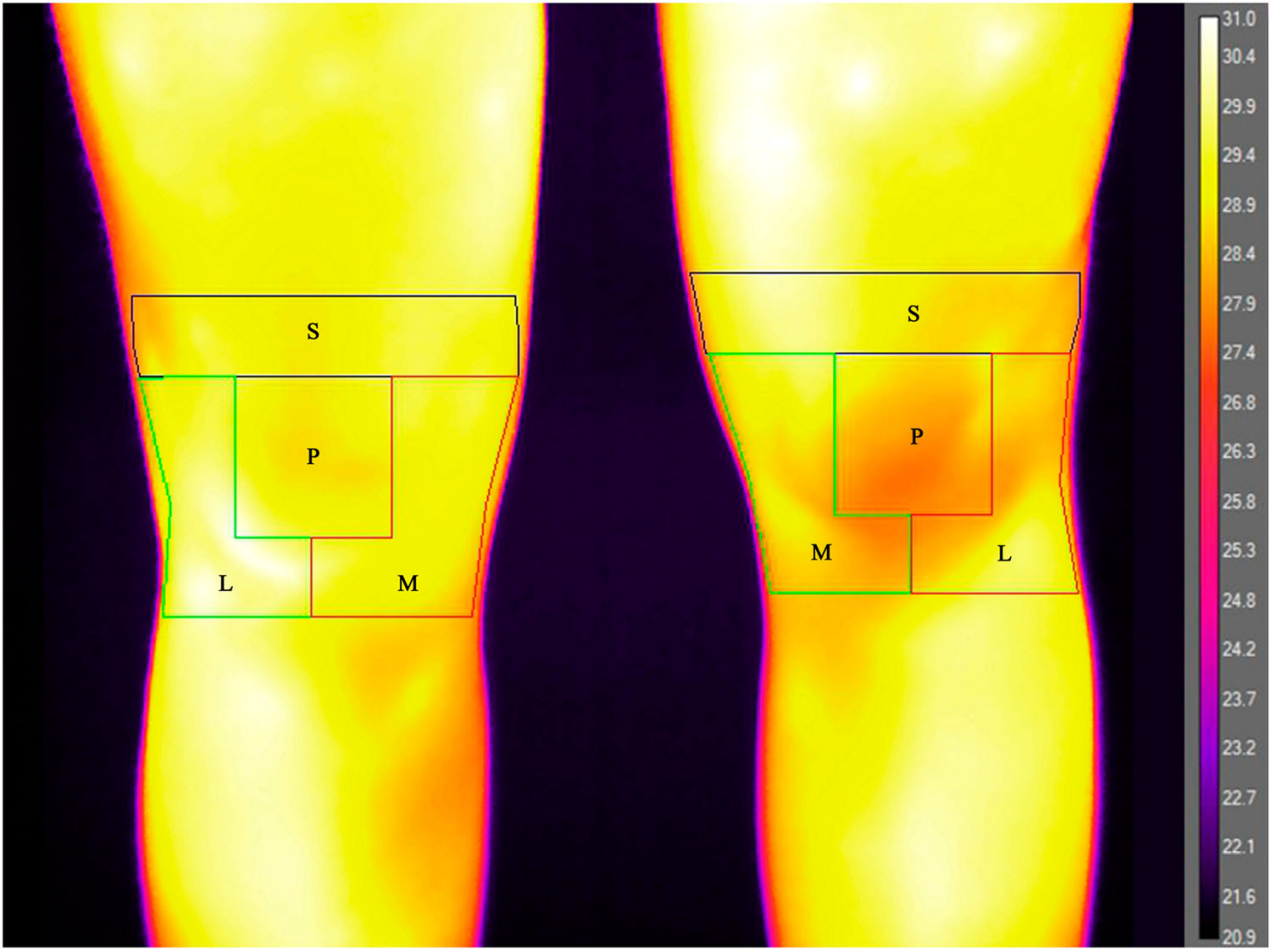
Twenty‐four‐year‐old female patient, 30 months after anterior cruciate ligament reconstruction (Tegner 7, 93 IKDC): the treated knee (left image) presents a higher temperature compared to the contralateral healthy knee. Both knees were analysed through the same regions of interest: L, lateral; M, medial; P, patella; S, suprapatellar. IKDC, International Knee Documentation Committee.

The mean temperature of the total joint was 31.4 ± 1.4°C for the ACL‐R knees and 31.1 ± 1.6°C for the healthy knees. The mean temperature of the total knee was significantly higher for the ACL‐R knee compared to the healthy knee (*p* = 0.010) (Figure 1). Analysing the mean temperature of the different areas, the mean temperature of the ACL‐R knee was significantly higher compared to the healthy knee for the patellar area (31.0 ± 1.6°C vs. 30.7 ± 1.8°C; *p* = 0.005), the lateral area (31.2 ± 1.2°C vs. 30.9 ± 1.4°C; *p* = 0.016) and the medial area (31.5 ± 1.5°C vs. 31.2 ± 1.7°C; *p* = 0.014), while no statistically significant differences were found for the suprapatellar area (31.6 ± 1.4 vs. 31.5 ± 1.5; n.s.).

**Figure 2:**
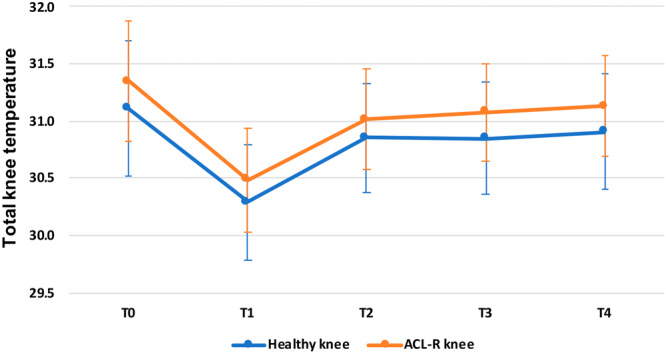
Mean total knee temperature in response to exercise for the anterior cruciate ligament reconstruction (ACL‐R) knee and the contralateral healthy knee at T0 (baseline), T1 (immediately after the exercise), T2 (after 5 min), T3 (after 10 min) and T4 (after 20 min). Presented as mean values and 95% CI of the mean. CI, confidence interval.

The analysis of the response to the exercises showed a significant reduction of the total knee temperature of the ACL‐R knee from the baseline value of 31.4 ± 1.4 to 30.5 ± 1.2 at T1 (*p* < 0.0005). After 5 min (T2), the total knee temperature of the ACL‐R knee significantly increased compared to T1 (31.0 ± 1.2; *p* < 0.0005), returning to values comparable to baseline (n.s.). After 10 and 20 min (T3 and T4), the total knee temperature remained stable compared to T2 (n.s.) and with values comparable to baseline (n.s.). The analysis of the four ROIs showed similar trends for all areas, as reported in Table 2. A similar trend for the total knee temperature and the four ROIs has been observed for the contralateral healthy knee, as reported in Figure 2.

**Table 2:** Temperatures of the evaluated knee areas at baseline and after exercise.

	**Area**	**ACL‐R knee**	**Healthy knee**	***p* Value**
T0 (baseline)	Total knee	31.4 ± 1.4^b^	31.1 ± 1.6^b^	0.010
	Patella	31.0 ± 1.6^b^	30.7 ± 1.8^b^	0.005
	Lateral	31.2 ± 1.2^b^	30.9 ± 1.4^b^	0.016
	Medial	31.5 ± 1.5^b^	31.2 ± 1.7^b^	0.014
	Suprapatellar	31.6 ± 1.4^b^	31.5 ± 1.5^b^	n.s.
T1 (post‐exercise)	Total knee	30.5 ± 1.2^a^	30.3 ± 1.4^a^	0.012
	Patella	30.2 ± 1.4^a^	29.9 ± 1.6^a^	0.008
	Lateral	30.3 ± 1.1^a^	30.1 ± 1.2^a^	0.049
	Medial	30.6 ± 1.3^a^	30.4 ± 1.4^a^	n.s.
	Suprapatellar	30.7 ± 1.2^a^	30.6 ± 1.2^a^	n.s.
T2 (5 min)	Total knee	31.0 ± 1.2^b^	30.8 ± 1.3^b^	0.032
	Patella	30.7 ± 1.4^b^	30.4 ± 1.6^b^	0.002
	Lateral	30.6 ± 0.9^a^,^b^	30.5 ± 1.1^a^,^b^	n.s.
	Medial	31.0 ± 1.3^a^,^b^	30.8 ± 1.4^a^,^b^	0.003
	Suprapatellar	30.5 ± 1.3^b^	30.6 ± 1.1^b^	n.s.
T3 (10 min)	Total knee	31.1 ± 1.1^b^	30.9 ± 1.4^b^	0.006
	Patella	30.8 ± 1.4^b^	30.4 ± 1.6^b^	0.001
	Lateral	30.7 ± 1.2^a^,^b^	30.6 ± 1.4^b^	n.s.
	Medial	31.0 ± 1.3^a^,^b^	30.7 ± 1.5^a^,^b^	0.012
	Suprapatellar	31.7 ± 1.1^b^	31.6 ± 1.2^b^	n.s.
T4 (20 min)	Total knee	31.1 ± 1.8^b^	30.9 ± 1.4^b^	0.006
	Patella	30.9 ± 1.4^b^	30.4 ± 1.7^b^	0.001
	Lateral	30.8 ± 1.0^a^,^b^	30.6 ± 1.2^b^	n.s.
	Medial	31.1 ± 1.3^a^,^b^	30.8 ± 1.5^a^,^b^	0.012
	Suprapatellar	31.7 ± 1.9^b^	31.6 ± 2.0^b^	n.s.

*Note*: T0 (baseline), T1 (immediately after the exercise), T2 (after 5 min), T3 (after 10 min) and T4 (after 20 min).

Abbreviation: ACL‐R, anterior cruciate ligament reconstruction.

^a^ *p* < 0.05 compared to T0.

^b^ *p* < 0.05 compared to T1.

The analysis of the response to the exercise at different times confirmed the higher temperatures in ACL‐R knees compared to the contralateral healthy knees. Moreover, the ACL‐R knees had a higher mean total temperature at all evaluations post‐exercise compared to the healthy knee at T1 (30.5 ± 1.2 vs. 30.3 ± 1.4; *p* = 0.012), T2 (31.0 ± 1.2 vs. 30.8 ± 1.3; *p* = 0.032), T3 (31.1 ± 1.1 vs. 30.9 ± 1.4; *p* = 0.006) and T4 (31.1 ± 1.8 vs. 30.9 ± 1.4; *p* = 0.006). More details on the mean temperatures of the four ROIs at the different evaluations post‐exercise are reported in Table 2.

**Figure 3:**
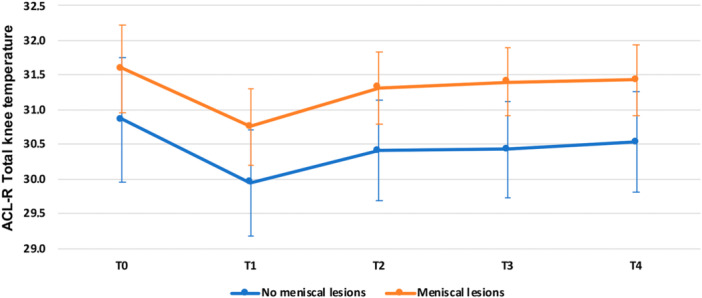
Mean total knee temperature in response to exercise for anterior cruciate ligament reconstruction (ACL‐R) knees with concomitant meniscal lesions and ACL‐R knees without concomitant meniscal lesions at T0 (baseline), T1 (immediately after the exercise), T2 (after 5 min), T3 (after 10 min) and T4 (after 20 min). Presented as mean values and 95% CI of the mean. CI, confidence interval.

The concomitant treatment of meniscal lesions at ACL‐R time was shown to influence the total knee temperature of the ACL‐R knees after exercise (Figure 3). Although there were no significant differences at baseline and T1 (all *p* = n.s.), ACL‐R knees with concomitant meniscal treatment showed higher total knee temperatures compared to ACL‐R knees without concomitant meniscal treatment at T2 (30.3 ± 1.1 vs. 31.3 ± 1.2; *p* = 0.047), T3 (30.4 ± 0.9 vs. 31.4 ± 1.1; *p* = 0.027) and T4 (30.5 ± 1.0 vs. 31.4 ± 1.2; *p* = 0.048).

## DISCUSSION

The main finding of this study is that the temperature of asymptomatic knees previously treated with ACL‐R is higher than that of the contralateral healthy knee. The temperature response to exercise is similar between ACL‐R knees and healthy knees, although the temperature remains higher in ACL‐R knees, and the presence of concomitant meniscal lesions further increases the temperature after the exercise. These results could indicate an inflammatory state persisting years after the ACL surgery, which could predispose patients to the early onset of knee OA.

The use of infrared thermography for the assessment of musculoskeletal diseases is increasing due to the ease of assessing the temperature of a specific body region [37, 51]. In recent years, infrared thermography has been proposed in clinical research as a new method to assess joint inflammation in several joints including the knee [13, 14, 15]. Articular inflammation also plays a role in patients undergoing ACL‐R surgery. Several studies documented an increase in biomarkers of inflammation and cartilage degradation products following ACL‐R [7, 34]. After the procedure, joint inflammation increases immediately and, although concentrations of proinflammatory cytokines begin to decrease 4 weeks later, they do not return to normal preoperative levels after the first month [27, 34]. This inflammatory state could be detected not only in the first post‐operative period but even years later. Larsson et al. [38] found an increase in proinflammatory cytokines in synovial fluid up to 5 years after reconstruction, demonstrating a persisting joint homoeostasis alteration following ACL‐R surgery.

The main aim of ACL‐R is to restore the biomechanics of the knee, since restoring normal kinematics reduces aberrant loading and stress on the joint structures [3, 28, 53]. Nevertheless, some studies reported partial restoration, while others observed continued persistent abnormalities, especially in terms of tibia position and rotation [2, 33]. Biomechanical alterations can cause increased stress to the cartilage layer, resulting in the production of proinflammatory cytokines and cartilage degradation products [5, 44]. In a study by Pietrosimone et al. on ACL‐R patients, the altered joint load has been linked to higher levels of degenerative enzymes and proinflammatory cytokines [44]. In another study by Erhart‐Hledik et al., ACL‐R patients showed how after exercise the levels of cartilage degradation products were similar to those of patients with OA, hence suggesting an alteration in the metabolic activity of chondrocytes following physical exercise [17]. Furthermore, biomechanical alterations after ACL‐R have been found not only in the tibiofemoral but also in the patellofemoral joint [62]. These altered patellofemoral joint loads can lead to cartilage thinning and softening, thus making it more susceptible to degeneration [12, 35]. This constitutes an unfavourable combination that could play a role in initiating or accelerating joint degenerative processes [49]. Therefore, the prolonged inflammatory state after ACL‐R could contribute to the cartilage degeneration observed following ACL‐R and promote the progression of OA [16, 39, 48, 55]. In this scenario, the early identification of the presence of inflammation and joint degeneration in asymptomatic patients previously treated with ACL‐R could provide useful information, and infrared thermography can represent a valid tool to indirectly detect joint inflammation in these patients.

The current study demonstrated that the temperature of knees previously treated with ACL‐R assessed with infrared thermography is 0.3°C higher than the contralateral healthy knees. It is known that the human healthy body tends to thermal symmetry and some preliminary studies reported asymmetries of 0.3°C as significant, even though the clinical relevance of this difference has yet to be established [24]. In this light, the higher temperature over two years after surgery of knees previously treated with ACL‐R is of particular interest, as it could underline persisting homoeostatic alterations even in a selected group of patients who did not exhibit any symptoms and had returned to their sports activities after the surgery. Despite the absence of symptoms in the operated knee, the higher temperature compared to the healthy contralateral knee supports the presence of an underlying inflammatory state, although not clinically detectable. In fact, all evaluated ACL‐R knees had an IKDC objective score graded as A, besides presenting no symptoms and allowing patients to perform their functional activities. While the increase in skin temperature of the knee could support the hypothesis of an underlying inflammatory state, future research should confirm this aspect.

The temperature difference was also confirmed in response to physical exercise, which could provide interesting information on the behaviour of operated joints after physical activity. The current study demonstrated that the skin temperature of knees with ACL‐R had a similar response to exercise compared to the healthy one after 2 min of exercise, with an initial decrease in temperature immediately following exercise, followed by a subsequent gradual return to the baseline temperature value, but always with higher temperature values. This trend confirmed the skin temperature response to the exercise already demonstrated in previous studies, due to redistribution of the blood circulation with consequent cutaneous vasoconstriction and vasodilation at the muscular level, which causes an initial reduction in skin temperature, followed by a slow return to the baseline values [14, 30]. In this regard, this response to exercise was found in a previous study on 13 healthy young volunteers (average age 25 years) [22]. The authors analysed the response to physical exercise using infrared thermography, showing a peak reduction in temperature between 2 and 3 min from the start of exercise and a subsequent gradual increase in temperature. While the trend over time in ACL‐R patients was similar to the healthy volunteers, some differences can be observed when comparing it with those found in 60 patients with knee OA (average age 61.4 years) [14]. In fact, while in the current study, the temperature of the ACL‐R and healthy knees returned to baseline values 5 min after the end of the exercise, the temperature of knee OA patients remained lower at the same time compared to baseline values. This difference between the studies could be justified by the different ages of the participants and the different activity levels performed, as well as by possible differences in the vascular response to exercise, and of the inflammatory and degenerative processes characterising ACL‐R patients from those with established OA, leading to different skin temperatures [18, 31, 36].

The analysis of the response to exercise in ACL‐R knees also demonstrated an influence of previously treated meniscal lesions on the knee temperature. Concomitant meniscal lesions further increased the skin temperature in ACL‐R knees after exercise. Patients with previously treated meniscal tears demonstrated higher skin temperatures at 5, 10 and 20 min after the end of the exercise. This could be related to the altered intra‐articular environment with consequent joint inflammation [43]. When the integrity of the meniscus is compromised, the contact pressure on the cartilage increases and the contact area decreases [10, 56]. Cartilage overload induces morphological, molecular and mechanical changes in cells and cartilage matrix leading to softening, fibrillation, ulceration and cartilage loss [6, 41]. It has also been shown that these biomechanical changes cause an alteration of the joint environment with the production of proinflammatory cytokines that contribute to further cartilage degeneration and structural damage [6, 23, 26, 32]. Furthermore, recent evidence suggests that the meniscus plays not only a biomechanical role but also a biological role [19, 54] through the increase in the production of proinflammatory mediators and enzymes capable of degrading the matrix [43]. This, together with the lack of cartilage protection, could be detrimental for the knee, especially during sporting activity due to the greater stress on the articular surface, which could favour joint inflammation and consequently early joint degeneration, as detected by the thermographic analysis [4, 8, 60].

This study presents some limitations. Although this is the first study evaluating the thermal pattern and response to exercise in knees previously treated with ACL‐R, the sample size is low and heterogeneous, and factors like sex and others may influence the obtained findings, and thus future studies with larger populations are needed to confirm the observed results. However, healthy contralateral knees were used as a reference, thus providing internal control and reducing the variability of the analysed sample. Moreover, the small patient cohort could impair the statistical power to detect the specific influence of different meniscal procedures on knee temperature. Future studies should better investigate these aspects and evaluate how the presence of previous meniscal lesions and their specific treatment can influence patients' knee temperature. Furthermore, objective and subjective clinical evaluations and knee laxity analysis with an accelerometer have been performed, showing an optimal clinical knee condition and good neo‐ligament retention, thus restricting the analysis to a homogeneous population with successful ACL‐R, where the detection of persisting changes is therefore even more meaningful. Another limitation is the lack of biomarker or imaging assessments, such as X‐rays and magnetic resonance, which can allow to directly evaluate the cytokines responsible for driving the inflammatory status of the ACL‐R knee and the presence of early degenerative changes. Future studies should confirm the usefulness of infrared thermography in detecting joint inflammation correlating its results with those obtained with biomarker evaluations or imaging analysis. Finally, the method of acquiring and evaluating thermographic images was based on previous literature, but nowadays, no method has been established as the gold standard in this field, and controversial results have been reported on the potential of this technology to properly document joint disease‐related findings, as in the study of Vargas et al. who reported no correlation between OA level and skin temperature [61]. It is possible that different settings, different cameras, lenses and devices may be more suitable for these evaluations in clinical practice. Therefore, future studies should help to further standardise the use of thermography for the evaluation of patients with ACL‐R to confirm its potential in identifying different patterns both in the research context and in clinical practice. This could have the potential of favouring better management of ACL‐R patient rehabilitation and return to sport to identify early degenerative joint changes and possibly to help develop and target treatments to interrupt inflammatory processes and early degenerative changes after ACL‐R surgery.

## CONCLUSIONS

This study demonstrated that the temperature of asymptomatic knees previously treated with ACL‐R is higher than that of the contralateral healthy knee. The temperature response trend to exercise is similar between ACL‐R knees and healthy knees, although the temperature remains higher in ACL‐R knees. The presence of concomitant meniscal lesions further increases the temperature after the exercise. These results could indicate an inflammatory state persisting years after the ACL surgery. Future studies should investigate the clinical relevance of these findings in terms of clinical outcomes and early onset of knee OA.

## AUTHOR CONTRIBUTIONS

Conceptualisation: Giuseppe Filardo. Methodology: Luca De Marziani, Simone Orazi, Luca Andriolo and Alessandro Di Martino. Data curation: Luca De Marziani and Angelo Boffa. Writing—original draft preparation: Luca De Marziani and Angelo Boffa. Writing—review and editing: Alessandro Di Martino, Luca Andriolo and Giuseppe Filardo. Supervision: Giuseppe Filardo and Stefano Zaffagnini. All authors have read and agreed to the published version of the manuscript.

## CONFLICT OF INTEREST STATEMENT

Stefano Zaffagnini reports non‐financial support from personal fees from I+SRL and grants from Fidia Farmaceutici SPA, Cartiheal Ltd., IGEA clinical biophysics, BIOMET and Kensey Nash outside the submitted work. The funders had no role in the design of the study; in the collection, analyses or interpretation of the data; in the writing of the manuscript; or in the decision to publish the results. The other authors declare no conflict of interest.

## ETHICS STATEMENT

The study was approved by the hospital Ethics Committee of the IRCCS Istituto Ortopedico Rizzoli, Bologna, Italy (protocol number 359/2023/Sper/IOR). Informed consent of all patients was obtained.
